# Does hyperlactatemia at admission predict mortality in acute medical patients? A population based cohort study

**DOI:** 10.1186/1757-7241-21-S2-A22

**Published:** 2013-09-09

**Authors:** Felix Haidl, Daniel Pilsgaard Henriksen, Mikkel Brabrand, Annmarie Lassen

**Affiliations:** 1 Department of Anesthesia, Sygehus Lillebælt, Kolding, Denmark; 2 Emergency Department, Odense University Hospital, Odense, Denmark; 3 Department of Medicine, Sydvestjysk Sygehus, Esbjerg, Denmark

## Background

An increased lactate level is related to elevated mortality in various subpopulations of critically ill patients, e.g. sepsis and trauma. The aim of the present study was to investigate to which degree lactate is related to increased mortality in a broad cohort of acute medical patients.

## Methods

Single centre cohort study. All adult patients admitted to the medical emergency ward of Odense University Hospital from March 2009 to August 2011, who had an arterial blood gas sample taken within six hours after admission were enrolled. Lactate was stratified in 1mmol/l (mM) intervals. Ten-day mortality after admission was assessed through the Danish Centralised Civil Registration system. A further stratification according to systolic hypotension (< 90 mmHg) was performed. Finally, a survival analysis (Kaplan-Meyer plot) was performed for the first ten days.

## Results

5,318 patients were enrolled, 2,493 male, median age 71 years (5% and 95% inter quartiles 25-91 years). Median lactate level was 1.2 mM (5% and 95% interquartile range 0.6-3.8 mM). Ten-day mortality was 382/5,318 (7.2 %). Ten-day mortality increased with increasing lactate at arrival with 79/1,778 (4.2 %) for lactate 0-0.99 mM, 132/2,182 (5.7 %) for lactate 1.0-1.9 mM, 71/614 (10.3 %) for lactate 2,0-2,9 mM, 29/174 (14.3 %) for lactate 3.0-3.9 mM, 23/87 (20.9 %) for lactate 4.0-4.9 mM, 6/42 (12.5 %) for lactate 5.0-5.9 mM, 10/18 (35.7 %) for lactate 6.0-6.9 mM, 7/11 (38.9 %) for lactate 7.0-7.9 mM and 25/30 (45.5 %) for lactate ≥ 8 mM (Cuzick’s test for trend, p < 0.001). This pattern was more marked in the hypotensive subpopulation. Survival analysis indicated that the increase in mortality was most pronounced within the first five days.

## Conclusion

Lactate levels drawn within six hours of admission is a predictor of mortality among patients admitted to the acute medical ward. Mortality is increased with each mM of lactate increase.

